# Fully online OSCEs: A large cohort case study

**DOI:** 10.15694/mep.2020.000214.1

**Published:** 2020-09-30

**Authors:** Anna Ryan, Aimee Carson, Katharine Reid, David Smallwood, Terry Judd

**Affiliations:** 1 University of Melbourne & University of Queensland; 2 University of Melbourne; 3 University of Melbourne & Austin Health

**Keywords:** Assessment, OSCE, Medical Education, Online, Remote, Clinical, Large Cohort, COVID, Pandemic

## Abstract

This article was migrated. The article was marked as recommended.

Objective Structured Clinical Examinations (OSCEs) are extensively used for clinical assessment in the health professions. However, current social distancing requirements (including on-campus bans) at many universities have made the co-location of participants for large cohort OSCEs impossible. While there is a developing literature on remote OSCEs, particularly in response to the COVID-19 pandemic, this is dominated by approaches dealing with small participant numbers.

This paper describes our recent large scale (n = 361 candidates) implementation of a remotely delivered 2 station OSCE. The planning for this OSCE was extensive and involved comprehensive candidate, examiner and simulated patient orientation and training. Our processes were explicitly designed to develop platform familiarity for all participants and included building on remote tutorial experiences and device testing. Our remote OSCE design and logistics made use of using existing enterprise solutions including videoconferencing, survey and collaboration platforms and allowed extra time between candidates in case of technical issues. We describe our process in detail including examiner, simulated patient, and candidate perspectives to provide precise detail, hopefully assisting other institutions to understand and adopt our approach.

Although logistically complex, we have demonstrated that it is possible to deliver a remote OSCE assessment involving a large student cohort with a limited number of stations using commonly available enterprise solutions. We recognise it would be ideal to sample more broadly across stations and examiners, yet given the constraints of our current COVID-19 impacted environment, we believe this to be an appropriate compromise for a non-graduating cohort at this time.

## Introduction

Objective Structured Clinical Examinations (OSCEs) are one of the most widely used forms of clinical assessment in the health professions ( Harden, Lilley and Patricio, 2015). Although their delivery varies across contexts, OSCEs typically involve candidates being assessed against defined criteria for competence as they move through a circuit of time-limited stations ( Khan *et al.*, 2013b). The use and movement of students through multiple stations, while necessary to improve sampling and standardisation, can be logistically complex ( Boursicot and Roberts, 2005; Khan *et al.*, 2013a). This is particularly true when large cohort of students are being assessed, requiring the involvement of numerous patients (simulated or real), examiners and invigilators.

Managing all the necessary participants and processes is difficult enough when they are all co-located and would, at first glance, seem impossible if they were not. However, the COVID-19 pandemic has necessitated a rapid and substantial rethinking of the way OSCEs are delivered and several recent papers have described potential solutions. One of the first of these, by Boursicot and colleagues (2020), adopts a minimal change approach by continuing to co-locate participants while employing social distancing and strict infection control measures. They were able to successfully implement this approach in a 56-candidate OSCE earlier in 2020. However, as the impact of the pandemic has deepened, the viability, acceptability and even the legality of co-locating participants has ruled out similar approaches for now.

A solution to the need to co-locate participants was provided as far back as 2008 by Wilkinson and colleagues ( Wilkinson *et al.*, 2008), who described the use of videoconferencing to connect remotely located candidates with a co-located group of examiners (their assessment format also used written scenarios rather than simulated patients). Videoconferencing has obviously come a long way since the Wilkinson study, and is now commonplace, affordable and accessible to most. Most of the recent reports of ‘remote’ OSCE implementations involve the use of one videoconferencing platform or another (e.g. Palmer *et al.*, 2015; Craig, Kasana and Modi, 2020; Lara *et al.*, 2020; Major *et al.*, 2020; Qatar University, 2020) with a relatively high degree of success. What they are missing however, is scale. Of the examples we reviewed, none involved a large number of candidates (i.e n > 100), which poses the question how and even if it would be possible to conduct a large scale OSCE in which none of the numerous participants are co-located.

In this paper we outline our successful implementation of a 2-station OSCE involving 361 candidates over 2 days using videoconferencing and other online collaboration tools. We believe that others, who may have discounted the possibility of delivering OSCEs during COVID-19 enforced lockdowns, may benefit by studying and adapting our approach during the coming months.

## Case Study Context

The University of Melbourne Doctor of Medicine (MD) program is a four-year graduate-entry medical program. Year 1 is a predominantly campus-based and pre-clinical year followed by three clinical years located in hospital and general practice settings (including a six-month research project in final year). There are approximately 350 students at each year level and OSCEs are key element of the assessment program during the first three years of the program.

Our usual multi-station OSCE processes involve large groups of students being held and monitored in a pre-exam waiting rooms, with smaller groups of students being shepherded to the site of the OSCE, being rotated through the available stations, and then accompanied to the post-exam waiting room. In addition to the large numbers of students involved, each individual OSCE station involves an examiner, simulated patient (or for some examination stations a real patient volunteer), and a number of invigilators. We generally run between 25-30 concurrent stations at any one time, and typically, two to five different OSCE stations are delivered at any one site in a single day (depending on site, station complexity and logistic considerations).

Our normal processes involve examiners engaging in an online training module in the lead up to each OSCE. They receive documentation about the station they will examine a few days beforehand, and attend a station briefing just prior to the OSCE. Examiners mark their station using a bespoke iPad app. Our marking rubrics include both checklists and global rating scales, and allow examiners to select from a range of predetermined comments that form part of individualised automated feedback reports distributed to every candidate a few days after the assessment.

Simulated patients attend a training session (usually conducted by the relevant station writer) a few days before the OSCE. Invigilators prepare the physical environment for the OSCE (which typically takes place across a series of outpatient or tutorial rooms) the day before, finalising and coordinating their arrangements during their pre-OSCE briefing.

As part of our normal quality assurance processes, feedback from students, simulated patients and examiners is collected via a paper or online survey. Incident reports forms are provided to all examiners in case of any disruption to any individual assessment. A small number of experienced examiners act as reserve examiners. If the reserve examiners are not required to examine, they act as quality assurance examiners, observing a selection of stations and examiners in order to advise on possible improvements to the station and the conduct of the examination more generally.

Our medical schools’ assessment strategy in response to the COVID-19 pandemic focuses on five key elements: equity, access, learning, pacing and certification. In summary: all students must have the opportunity to cover relevant content prior to each assessment, all students must be able to access and engage with our assessments, assessments that promote and guide learning are prioritised, and wherever possible we avoid delaying assessments. Assessments are aligned with subject and course outcomes and we engage in rigorous quality assurance processes. Our central aim is to assure stakeholders that our students have requisite knowledge and skills to be able to provide safe clinical care upon graduation.

## Remote OSCE Preparations

In year 2 of our program we deliver two OSCE stations in the middle of the year, another two in the middle of the second semester, and five at the end of the year. Our overarching plan was to remotely deliver this two-station OSCE, over two days, with all participants undertaking the OSCE from their own home or workplace using video conferencing and other online or mobile technologies. We decided on the combination of Zoom for video conferencing, Qualtrics for examiner marking, Microsoft Teams for invigilator administration, and texting and/or phone calls for communication between invigilators and examiners. Our university has enterprise licences for Zoom, Qualtrics and Teams, which clearly influenced our choice of technologies. However, alternative platforms could fulfil similar roles if required.

Given the number of uncertainties involved in switching from in-person to remote delivery - with concerns about the reliability, timing and logistical complexity of a video-conferenced OSCE being foremost - we elected to deliver only one station per day. As a result, our two- station OSCE was delivered over two successive days. In an obvious concession to remote delivery, which also reflected the socially-distanced teaching and learning opportunities available to our students in the lead up to the OSCE, both stations were clearly focused on history-taking skills. In essence, each station functioned as a three-way telehealth consultation between the student and a simulated patient; facilitated by an observing examiner.

Our planned assessment blueprint was reviewed to ensure all students had received the requisite teaching and learning opportunities prior to being assessed in this OSCE. Given the significant disruption to their learning experiences, we changed the focus from rotation-specific topics to more foundational and core topic content of relevance and importance to all students no matter which rotating term they had undertaken. Of note, this rotation-specific learning will be covered in supplementary teaching and learning activities. Delivery of our remote OSCE occurred a few weeks later than scheduled to allow for testing of the delivery approaches and platforms and to provide essential hands-on training and experience for online examiners and invigilators.

The two OSCE stations were developed, reviewed and refined by members of our Clinical Assessment Review Panel. In the lead up to the assessment, they were modified to ensure the script and presentation were consistent with the COVID-19 pandemic situation and current government advice on social distancing. For example, the patient in one scenario recently underwent a COVID-19 swab for worsening cough and had not had contact with anyone outside her home as she had been correctly abiding by current social distancing requirements.

### Student Orientation and Training

Through parallel work on “written” computer-based assessment with remote invigilation we had previously ensured all 1400 medical students in the school had access to an appropriate device with videoconferencing (Zoom) functionality. We had also implemented a range of remote case-based learning activities, including modified mini-CEX assessments, with clinical teachers via Zoom - students were therefore already quite familiar with the platform and online assessment with a remote examiner. Students were informed that the remote OSCE would have a number of similarities to these mini-CEXs.

We developed a remote OSCE student guide leveraging the content from the existing remote “written” assessment guide. This supplemental guide outlined the examination process - number of examiners; possible presence of a quality assurance observer; duration of the station (eight minutes including a question from the examiner at seven minutes); importance of appropriate professional behaviour and maintaining academic integrity. As with our written examination guidelines, students were required to complete their examination in a quiet, private and well-lit room. They were explicitly advised that they were not to access any course materials during the OSCE, but were allowed to access a sheet of blank paper and a pen for taking notes during the examination. Students were advised to test their network, device and Zoom functionality prior to the examination and prepare for any likely contingencies (e.g. ready access to a charger for those using a laptop or tablet).

### Examiner Orientation and Training

Examiners were oriented to the remote OSCE format by describing it as similar to a three-way telehealth consultation. They were advised that the assessments would be completed online and could be undertaken on the same device they used for Zoom or on a different preferred device. Any device capable of supporting Zoom and with an internet browser was suitable (i.e. including smartphones).

Examiners were encouraged to engage with our usual online OSCE training module. Our normal process for creating OSCE stations involves the use of a bespoke iPad application and record management system (see Judd *et al.*, 2017). We used the Qualtrics survey platform for our remote OSCE and were able to create marking rubrics with (more or less) equivalent functionality and security to our bespoke system. As part of their training, examiners were provided with access to a sample OSCE station marking rubric on Qualtrics, allowing them to interactively watch and mark a video simulation of the station. They were also provided with instructions on how to set up an online timer that they could use during both the training and the remote OSCE.

All examiners were invited to attend one or more mock remote OSCEs in the lead up to the real examination. The mock OSCEs involved examiners and other staff playing the roles of student, patient, examiner and invigilator to enhance familiarity with the roles, processes and structure of the upcoming OSCE. Examiners were provided with detailed information about the OSCE stations via email in the days leading up to the examination.

### Simulated Patient Orientation and Training

Simulated patients were recruited from our usual pool of professional actors with the additional requirement that they have an appropriate device and reliable internet access. There were asked to hold a Zoom test meeting to evaluate internet reliability and connection and confirm essential Zoom functionality and familiarity prior to committing to our remote examinations. Our usual simulated patient training session was delivered two weeks before the OSCEand simulated patient mobile numbers were required as a backup in case of any technical difficulties.

## Remote OSCE Design & Logistics

Our goal was to create an OSCE experience that would involve similar logistics to our face-to-face OSCEs, enacted over video rather than in-person and with the use of virtual rather than physical rooms. Our cohort of 361 students was divided among five Zoom streams (meetings), each containing five exam rooms (i.e. five examiners and five simulated patients per stream), so that at any given time there were 25 students being examined.

### Student Experience

**Figure 1.**
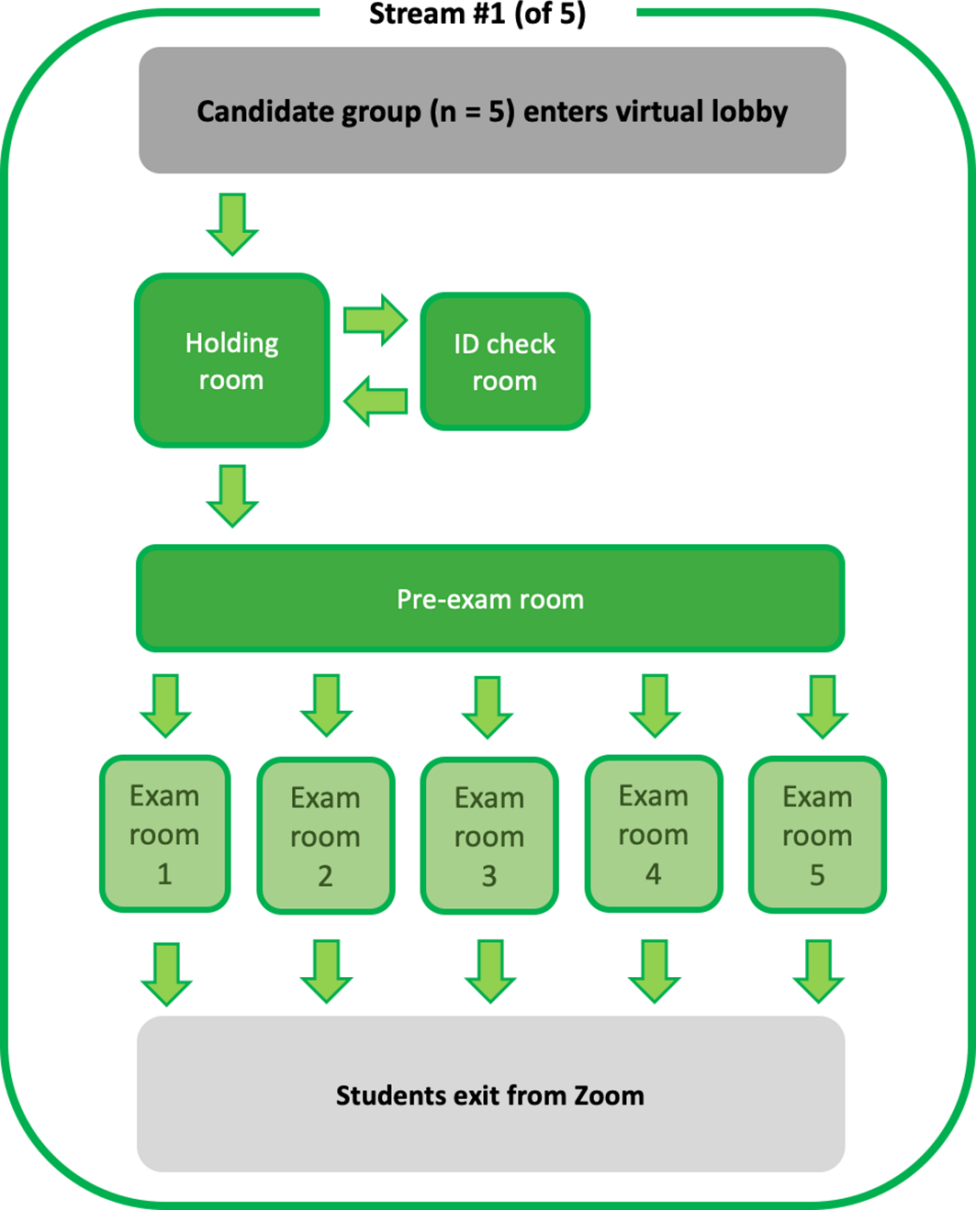
Representation of the virtual room in the remote OSCE.

Figure 1 describes the flow of students through one of our five Zoom streams. Each candidate joining their nominated Zoom stream entered via a **virtual lobby.** They were not connected to any other participants at that stage and were required to wait there and until the meeting host manually admitted them to the OSCE **holding room**. The **holding room** (the main Zoom meeting room) served a similar purpose to the registration room (or “in-room”) in our normal OSCEs, with candidates being registered and briefed about how the remote OSCE would be run. All other rooms within the remote OSCE stream were created as breakout rooms. From the holding room, each candidate was admitted in turn to the **ID check room** where they were met privately by an invigilator to verify their identity (ID) and their immediate environment scanned (using their webcam) to confirm that there were no unauthorised persons or materials present. From there they were moved back into the holding room then once all candidates in their group had had their IDs checked all were moved to the **pre-exam room**. At this point an invigilator provided the candidates with the station prompt (equivalent to what would be placed on examination room doors during our normal OSCEs) by sharing their screen. After a one-minute reading time, each candidate was then moved to one of the 5 **exam rooms** where a simulated patient andexaminer were already in place. In contrast to our normal OSCEs, there was no virtual equivalent of an “out-room”, with candidates exiting the Zoom stream directly from their exam room at the completion of their assessment.

### Scheduling

Groups of 5 candidates were scheduled to enter each Zoom stream at 15-minute intervals. As one group of candidates was being examined, then next group was progressing through the holding room and having their ID and environment checks completed. A 20-minute break was scheduled after each five completed groups.

The duration of each station was 8 minutes. The 15-minute interval between groups therefore nominally equates to a 7-minute break between candidates for examiners, which is five more minutes than we would normally allow but was deemed necessary to allow for delays in admitting and moving candidates between breakout rooms and for interruptions to individual assessments due to loss or breakdown in video stream quality to any of the participants. Based on our use of 5 concurrent Zoom streams, each with 5 virtual exam rooms and these timings, the nominal time required for all candidates (n=361) to complete a single station OSCE (excluding the abovementioned 20-minute breaks) was 3 hrs and 45 minutes. This compares with approximately 2 hrs and 30 minutes to complete an equivalent face-to-face single station OSCE involving 25 examiners. In reality, the actual running time on the first day of our remote OSCE, again excluding breaks, was closer to 4 hrs and 30 minutes, or 45 minutes over schedule. The process ran noticeably smoother on the second day and was only about 20 minutes over schedule.

### Examiner and Simulated Patient Perspective

Observers, examiners, invigilators and simulated patients were asked to enter their allocated Zoom stream approximately 50 minutes before the designated examination start time. One examiner in each stream (the lead examiner) then briefed the remaining four examiners on the station content and checked that everyone was comfortable with the technical and logistic aspects. Examiners were encouraged to have paper copies of the marking rubric on hand in case of any technical issues with the Qualtrics marking platform and all were asked to keep a written running tally of the station score and the overall rating for each student as a failsafe backup measure. Once the examiner briefing was complete, examiners were moved into their designated **exam room** to meet with their simulated patient where they remained throughout the OSCE.

Examiners were notified by text by the **pre-exam room** invigilator when reading time had commenced for each new group of candidates, allowing them to ensure that they and their simulated patient were ready to proceed. Follow admission of a new candidate to their exam room, the examiner then verified their name and ID, asked them to commence the exam, disabled their own audio and video, and started the exam timer. Examiners then assessed each candidate using the provided Qualtrics marking rubric. At the seven-minute mark of each exam, examiners enabled their audio and video and asked their candidate a question about the case. At the eight-minute mark examiners then informed their candidate that the exam was complete and asked them to leave the Zoom meeting. Examiners then texted the pre-exam invigilator a text message to notify them that their assessment was complete, to communicate any problems that they had encountered during the assessment and to confirm that they were ready to receive their next candidate.

### Moderator and Invigilator Perspective

Each Zoom stream employed four invigilators, each fulfilling a different role:

• Zoom moderator and roving invigilator (meeting host)
• Holding room invigilator
• ID and environment check room invigilator
• Pre-exam room invigilator (co-host)

The Zoom moderator hosted the stream and was responsible for moving participants into and between rooms as required. The pre-exam room invigilator (co-hosts) was able to move between virtual rooms and could serve as a reserve host if the moderator experienced technical issues. Invigilators communicated with each other during the OSCE via MS teams and the pre-exam room invigilators communicated with examiners about their readiness to progress to the next group and to triage technical and logistical issues via text messaging. Zoom’s chat feature was disabled to prevent communication between candidates while in the holding and pre-exam rooms.

From the invigilators’ perspectives, the process for each group of candidates within a stream involved the moderator moving a group of students from the ‘lobby’ into the **holding room** at their allocated time. The holding room invigilator supervised students within this room while the moderator moved individual students to and from the **ID check room** - where another invigilator was responsible for verifying student ID and scanning the student’s examination environment. Each student was moved back into the **holding room** by the moderator once the check was completed. Once the ID/environment check for all five students in that group was completed the moderator moved the entire group into the **pre-exam room**. The pre-exam room invigilator reminded students of the timing of the exam, reiterated the academic integrity requirements, and indicated that they must leave the stream immediately at the completion of the exam. At this point, the invigilator shared their screen to allow students to read the station prompt. The pre-exam room invigilator then sent a text message to the five examiners in their stream to notify them that their next candidate would be arriving shortly. In response, the invigilator received a text from the examiner once each current candidate’s assessment was completed. If a message from the examiner was not received, the pre-exam invigilator would enter the exam room after 10-12 minutes ascertain if there were any problems and to confirm that the examiner and simulated patient were ready to receive their next candidate.

## Reflections on our Experience

Much to our collective relief, there were no major logistical dramas during our first experience of remotely delivered OSCEs. There were minor disruptions to a couple of simulated patients’ internet functionality. An examiner also lost connection altogether during an exam at one point but, serendipitously, was being monitored by an experienced quality assurance observer who was able to step in, take over the examiner’s role and complete the exam. We experienced very few issues with student devices and connectivity, which is perhaps unsurprising all had been engaging in regular Zoom-based tutorials in the weeks leading up to the remote OSCE.

Our formal evaluation process sought feedback from both students and those involved in delivering the OSCE. Overall, the feedback from examiners, observers and simulated patients was very encouraging. All agreed that the OSCE was well organised and executed, and almost all agreed that they were comfortable working in the online environment. Reassuringly SPs managed the online process well, with examiners rating their representation of real patients highly. All SPs indicated that they were prepared to undertake an online OSCE in the future. Similarly, all examiners reported that their training prepared them well to examine and all indicated they were prepared to examine online again. Invigilators felt the process closely resembled the face-to-face OSCEs and agreed that participation in the mock remote OSCE better equipped them for the real remote OSCE. Informal feedback from examiners also suggested that the OSCE stations we developed effectively simulated a telehealth consultation, supporting the face validity of our assessment design. However, a number of examiners suggested that the remote OSCE involved a substantially higher cognitive load than for face-to-face OSCEs, due to having to manage multiple windows/screens, text messages from invigilators, back up marking sheets and student allocation/timing details. However, some of this additional load is likely have been temporary, as examiners who were involved in both remote OSCE days reported the second day was easier, and examiners who had attended the preparatory mock remote OSCE reported fewer issues or concerns.

Student feedback, while less positive than from other OSCE participants, was nonetheless reassuring about the logistic aspects. The majority of students believed that the OSCE was well organised and executed and that the online environment worked well for the OSCEs. It should be noted that student opinion was mixed as to whether they felt they could demonstrate their skills in this format. Some students also raised concerns about the consistency of timing of the station interactions and our use of examiner controlled individual timers is quite a change from the normal process of coordinated synchronous timers. Students appreciated the reduced waiting time in the online OSCE (compared with face-to-face) and the convenience of being at home. Some students did, however, suggest that there was too little information about the OSCE process and what to expect on the day. Nonetheless, despite some student concerns, analysis of these OSCEs suggests that their statistical characteristics (distributions, average scores, cut scores) were highly comparable to similar history-taking OSCEs last undertaken face-to-face.

The organisational logistics of our large-cohort remote OSCE were complex, but achievable in our context. We delivered two stations in two days, and this allowed structured assessment of our students on two history-taking stations. We purposefully selected core and important presentations to examine, but all students have only been assessed on two stations and only been examined by two examiners. We recognise it would be ideal to sample more broadly across stations and examiners, yet any increase in the number of stations would involve a huge and potentially unmanageable investment of time and personnel. Given the constraints of our current COVID-19 impacted environment, we believe this to be an appropriate compromise for a non-graduating cohort at this time. We do not currently have a viable OSCE-like solution for remote assessment of physical examination skills.

## Plans for Future Remote OSCEs/Lessons Learned

We are keen to explore the possibility of examining more than one station per day. An obvious way to reduce the overall time required to deliver each station is to reduce the ‘extra’ time allocated between candidates. We allowed seven minutes to cover marking time and technical and logistic contingencies versus only two minutes in our usual face-to-face OSCEs. Informal feedback from examiners suggested that while this 7 minutes was appropriate while familiarising themselves with the exam processes during their first few candidates it could have been reduced after that. Reducing it to four minutes, for example, could reduce our nominal per-station delivery time by approximately 45 minutes. However, reducing the between candidate time allocation also increases the likelihood that a technical or logistic issue occurring in one of the exam rooms might not be resolved in time to place the next group of students. That is, a delay in one exam room inevitably leads to a delay across all exam rooms in that stream, which occurred on a number of occasions during the first day of our remote OSCE even with the full 7-minute time allowance in place.

An alternative solution could be to allocate students to exam rooms based on exam room availability/readiness rather than adhering to a strict schedule. That is, rather than being allocated as a group, individual students are placed in an exam room as soon as it becomes available. This would require a number of changes to our existing processes - for example, students would be placed in a queue rather than in discrete groups, and examiners would provide individual students with the station prompt rather than viewing it as a group in the pre-exam room. In theory, adopting this type of model would minimise both the time required between candidates and the potential disruption to other examiners (and the overall exam timing) that can occur when an issue occurs in one exam room.

Our next mid semester two-station OSCE is imminent, and the key changes we have made involve additional communication to students around the flow of the OSCE (including Figure 1 in our remote OSCE student guide) and the timing arrangements (making it explicit that the examiners are keeping time and exactly when they start and end the timer).

## Conclusion

While admittedly logistically complex, we have demonstrated that it is possible to deliver a remote OSCE assessment involving hundreds of participants, none of whom are co-located, using commonly available videoconferencing, survey and collaboration platforms. We hope sharing our experience will help other health professions education programs continue their important work during these complex times, and that through sharing all our experiences we will be able to develop and build on a body of knowledge around an important and timely adaptation of the Objective Structured Clinical Examination.

## Take Home Messages

• Remote OSCEs with large cohorts are logistically complex but certainly possible with an extensive team of invigilators, simulated patients, examiners and observers/reserve examiners
• Familiarising students with the assessment format through clinical tutorials ensured device compatibility and reduced novelty of the new assessment
• Mock assessments provide valued opportunities for invigilators and examiners to rehearse and develop new workflows for managing the process
• Technical issues are inevitable, and ways to manage these (including extra time allowances) need to be built into planning
• “Overcommunication” about the process is advisable, particularly for students

## Notes On Contributors

**Anna Ryan** is Associate Professor and Director of Assessment, Department of Medical Education, Melbourne Medical School, University of Melbourne and Honorary Associate Professor, Office of Medical Education, University of Queensland. ORCID ID: https://orcid.org/0000-0001-7458-027X

**Aimee Carson** is Clinical Assessment Coordinator, Department of Medical Education, Melbourne Medical School, University of Melbourne.

**Katharine Reid** is Director of Evaluation and Quality, Department of Medical Education, Melbourne Medical School, University of Melbourne. ORCID ID: https://orcid.org/0000-0002-4600-8847

**David Smallwood** is Chair of the Clinical Assessment Review Panel, Department of Medical Education, Melbourne Medical School University of Melbourne & Director of The Department of General Medicine, Austin Health. ORCID ID: https://orcid.org/0000-0002-4719-8558

**Terry Judd** is Lead for Technology Enhanced Assessment, Department of Medical Education, Melbourne Medical School, University of Melbourne. ORCID ID: https://orcid.org/0000-0002-4692-6701
