# Supplementary material for: Fully online OSCEs: A large cohort case study
Source: MedEdPublish (2016). 2020 Sep 30;9:214. [Version 1] doi: 10.15694/mep.2020.000214.1 (PMC10699396; doi:10.15694/mep.2020.000214.1)
Supplement: Peer review history

# Reviewer report: Reviewer response for version 1

**DOI:** 10.21956/mep.20195.r31249

**Published:** 2020-12-16

**Authors:** Ken Masters

**Affiliations:** 1 Sultan Qaboos University

This review has been migrated. The reviewer awarded 3 stars out of 5 An interesting paper on running remote, online OSCEs with a large cohort of students. The paper is well-timed, and will, no doubt, provide very useful information to others wishing to undertake this. The full process is explained in appropriate detail.Among other things, the paper makes it clear that the process has to be extremely well-planned and co-ordinated, and, even with that, there will be problems. In addition, the process is limited to one station per day for two days, so scaling may be over-demanding for both students and staff.One area that I would like to see addressed in more detail, however, is the Results of the surveys. I realise that this is not a full research paper, but the authors do present results from a survey, and these are presented too vaguely. Proper statistics should have been provided.There are also a few small typos (e.g. “in a pre-exam waiting rooms,” )Nevertheless, a useful read for those needing to conduct OSCEs remotely.

Reviewer Expertise:

NA

No decision status is available

# Reviewer report: Reviewer response for version 1

**DOI:** 10.21956/mep.20195.r31250

**Published:** 2020-10-12

**Authors:** Mohamed Al-Eraky

**Affiliations:** 1 University of Dammam

This review has been migrated. The reviewer awarded 5 stars out of 5 This is a timely paper that address a hot issue these days. I agree with authors that most literature addressed control measures to manage OSCEs for small numbers, under certain precautions, but not fully online. Its’s wonderful to document your experience in MedEdPublish. I think there were many challenges, related to: examiners training on remote OSCE along with other logistics issues, such as: accurate timing of the virtual admission and departure of different stakeholders, namely: students, examiners and simulated patients, ID check and many others. This is a very interesting paper that readers would learn many lessons to plan and administer a fully-online OSCE. Excellent job! Good luck!

Reviewer Expertise:

NA

No decision status is available
